# Prospective study of canine leptospirosis in shelter and stray dog populations: Identification of chronic carriers and different *Leptospira* species infecting dogs

**DOI:** 10.1371/journal.pone.0200384

**Published:** 2018-07-11

**Authors:** Bruno Alonso Miotto, Aline Gil Alves Guilloux, Barbara Furlan Tozzi, Luisa Zanolli Moreno, Aline Santana da Hora, Ricardo Augusto Dias, Marcos Bryan Heinemann, Andrea Micke Moreno, Antônio Francisco de Souza Filho, Walter Lilenbaum, Mitika Kuribayashi Hagiwara

**Affiliations:** 1 Departamento de Clínica Médica, Faculdade de Medicina Veterinária e Zootecnia, Universidade de São Paulo, São Paulo, São Paulo, Brasil; 2 Departamento de Medicina Veterinária Preventiva e Saúde Animal, Faculdade de Medicina Veterinária e Zootecnia, Universidade de São Paulo, São Paulo, São Paulo, Brasil; 3 Departamento de Microbiologia e Parasitologia, Universidade Federal Fluminense, Niterói, Rio de Janeiro, Brasil; Cornell University, UNITED STATES

## Abstract

Dogs are highly susceptible to the leptospiral infection, notably stray and sheltered dogs. Unsanitary conditions often observed in dog shelters may predispose the introduction and spread of leptospires among sheltered populations, potentially increasing the chances for the inadvertent adoption of asymptomatically infected animals. The present work describes a longitudinal study using a multidisciplinary approach for the identification of chronically infected dogs and the characterization of potentially pathogenic strains circulating among stray and sheltered dog populations in São Paulo, Brazil. A total of 123 dogs from three populations were included. The initial evaluation consisted of blood and urine quantitative PCR testing (qPCR), the detection of specific antibodies by microscopic agglutination test (MAT), physical examination and hematological and serum biochemistry analyses. The qPCR-positive dogs were prospectively examined, and reevaluations also included culture from urine samples. Positive qPCR samples were subjected to 16S rRNA and *secY* gene phylogenetic analysis. The recovered strains were characterized by Multilocus Sequence Typing, polyclonal serogroup identification and virulence determination. Leptospiruria was detected in all populations studied (13/123), and phylogenetic analysis revealed that 10 dogs had *L*. *interrogans* infection. Three dogs (3/13) had *L*. *santarosai* infection. The *secY* phylogenetic analysis revealed that the *L*. *santarosai* sequences clustered separately from those obtained from other hosts. Ten leptospiruric dogs were reevaluated, and three dogs presented persistent leptospiruria, allowing culturing from two dogs. The strains were characterized as *L*. *interrogans* serogroup Canicola (virulent) and *L*. *santarosai* serogroup Sejroe (not virulent). Serum samples were retested by MAT using the DU92 and DU114 strains as antigens, and no increased seroreactivity was detected. Asymptomatic *L*. *santarosai* infection was observed in all populations studied, suggesting a possible role of dogs in the chain of transmission of this leptospiral species. The results suggest a genetic distinction between lineages of Brazilian *L*. *santarosai* maintained by dogs and other animal hosts. Our findings revealed that dogs could act as maintenance hosts for distinct pathogenic *Leptospira*, highlighting also that asymptomatically infected dogs can be inadvertently admitted and adopted in dog shelters, potentially increasing the risks of zoonotic transmission.

## 1. Introduction

Leptospirosis is a bacterial disease caused by pathogenic helical shaped spirochetes of the genus *Leptospira* [1]. Pathogenic *Leptospira* are currently classified into more than 250 serovars and ten genomospecies [2]. Virtually any mammalian species can be affected, and leptospiral infection can cause a broad spectrum of clinical manifestations, ranging from severe, life-threatening conditions to mild, self-limiting febrile illness and asymptomatic infections [1].

The disease is recognized as the most widespread zoonosis and has emerged as a major public health issue in much of the developing world [3]. Human leptospirosis is frequently observed in poverty-stricken populations living in tropical regions [4], and it is considered one of the major neglected diseases worldwide, notably in Latin America [5].

The transmission of the disease is strongly driven by environmental factors, such as high pluviometric precipitation rates, flooding, natural disasters, uncontrolled urban expansion and poor sanitation [6]. The exposure to water and soil contaminated by the urine of infected animals is the most common route of transmission to humans and domestic animals [6], and rodents are considered the major source for human infection, a role likely attributed to its synanthropic behavior and widespread distribution [3]. Nevertheless, recent One Health approaches have been used to circumvent crucial epidemiological aspects of leptospirosis, and several studies have pinpointed a significant role of different mammalian hosts in its zoonotic transmission [6–9].

Canine leptospirosis has been largely described [10,11], and the clinical presentation in dogs is often associated with *L*. *interrogans* and *L*. *kirschneri* infection [11]. Chronically infected individuals can persistently harbor leptospires without overt clinical signs, and dogs are referred to as reservoir hosts for pathogenic *Leptospira* [12–15], notably *L*. *interrogans* serovar Canicola [16], a pathogenic serovar that can infect humans and other mammals [17–19].

While the actual role of dogs in the zoonotic transmission of leptospirosis still remains poorly documented, and the overall contribution of dogs to the burden of human leptospirosis has yet to be determined [20], asymptomatic urinary shedding of leptospires among dog populations has been widely reported [12,14,21–25], thus indicating that dogs at the very least can contribute to the spread of pathogenic *Leptospira* into the environment.

Proper management of chronically infected dogs should be implemented to reduce environmental contamination; however, the identification of such individuals remains challenging. Renal carriage of leptospires is not necessarily associated with the presence of serum antibodies against *Leptospira* [26], restricting the use of serological tests to identify asymptomatically infected dogs. Culturing of leptospires, albeit essential to confirm infection, is also not a suitable technique for the identification of urinary shedders, especially for presenting frequent contamination, low sensitivity and fastidious growth of the pathogen [11]. More recently, the polymerase chain reaction (PCR) has emerged as the main diagnostic tool for the detection of leptospiruric dogs [14] and several PCR protocols have been developed to detect leptospiral DNA in canine urine samples [12,27]. However, the intermittent shedding of leptospires typically observed in maintenance hosts may lead to false-negative PCR results, and the identification of leptospiruric dogs based exclusively on a single PCR evaluation may restrict any considerations regarding the occasional, intermittent or persistent urinary shedding of the pathogen.

In face of these limitations, the identification of chronically infected individuals should rely on prospective studies using multiple laboratory tests, in order to provide clinical, laboratorial and serological data to fully characterize the dog’s carrier status [28]. More importantly, longitudinal studies may also potentially increase the chances of recovering leptospires in the culture media for appropriate characterization.

Stray dog populations and dogs kept under shelter conditions are considered more susceptible to the infection because of a higher degree of environmental exposure to pathogenic *Leptospira* [29,30]. High seroprevalence has been reported in these populations worldwide [30–34], which is a situation that might rise as a public health concern. In recent years, there has been a considerable increase in the number of dog shelters in Brazil, particularly in São Paulo state. This scenario comes as a result of the implementation of a local law (law n. 12.916, enacted on April 16, 2008), which has banned the euthanasia of stray dogs captured by animal control agencies, establishing sterilization and adoption as the main legal strategies for the control of stray and sheltered populations. These circumstances led to the emergence of several overpopulated shelters, often marked by poor sanitary conditions and high levels of rodent infestation. Many of these shelters experience structural deficiencies and limited funding, whereas sanitary admission or adoption protocols are frequently not implemented. Such conditions might represent increased chances of leptospiral transmission among housed dogs and occupational risks to kennel workers and caretakers [35,36], potentially increasing the chances for the inadvertent adoption of chronically infected animals, which would hypothetically contribute to dog-to-human transmission by bringing asymptomatic carriers closer to adopters and their households [15,35,37].

To promote evidence-based knowledge regarding asymptomatic urinary shedding of leptospires in dogs, the present study proposes the identification of chronically infected animals and the characterization of potentially pathogenic strains circulating among stray and sheltered dog populations in São Paulo, Brazil. To do so, we have designed a cross-sectional study to initially identify leptospiruric dogs, followed by a prospective evaluation using a multidisciplinary approach to characterize the chronic carrier state of the infected animals and to identify the infecting strains.

## Materials and methods

### Studied populations and environmental conditions

Dogs from three populations were included: (I) 24 out of 30 dogs kept in a public shelter from the city of Mogi das Cruzes, located in the eastern region of São Paulo State, Brazil; (II) seven stray dogs out of an estimated population of 32 individuals living on the University of São Paulo (USP) campus [38], located in the west region of São Paulo City, Brazil; and (III) 92 out of 103 dogs kept in a public shelter located on the USP campus.

#### Mogi das Cruzes shelter

Most dogs were housed individually or in groups of up to three animals. The stalls had natural light and were frequently cleaned. No accumulation of debris or major structural deficiencies were noticed during the sample collection. The local staff reported rare sightings of rodents, and pest control was carried out systematically. The animals had full veterinary support, although no vaccination protocols against leptospirosis were implemented at admittance or during the animal’s stay.

#### Stray dogs from the USP campus

Most of the stray dogs were community animals. Food was supplied sporadically by community members, and vaccination against leptospirosis and basic veterinary assistance were occasionally provided by veterinary school students.

#### USP shelter

The shelter was designed to house 40 dogs appropriately and 63 dogs were lodged under improvised conditions. Most dogs were housed in groups of two to 10 animals. At the time of sampling, the facility had several inappropriate structural features, such as broken roof tiles, water leakages, generalized accumulation of debris and no appropriate drainage of rain or collection of organic waste and sewage. Pest control was carried out only sporadically and signs of high levels of rodent infestation were present, such as bitten newspapers, droppings inside drawers and sightings of rodents during the day. Most of these conditions were properly remedied by the shelter administration after the study period. All dogs were sterilized before adoption, and the animals were frequently immunized against leptospirosis, although no systematized vaccination protocol was implemented.

### Study design and samples

The cross-sectional evaluation of the studied populations consisted of clinical evaluation (physical examination, serum biochemistry analysis and evaluation of hematological parameters), blood and urine quantitative PCR testing (qPCR) and the detection of serum anti-*Leptospira* antibodies. All qPCR-positive dogs were prospectively evaluated to confirm the persistence of infection and to try to recover viable leptospires for proper characterization. Reevaluations included leptospiral culture from urine samples, blood and urine qPCR testing, the detection of serum anti-*Leptospira* antibodies and clinical evaluation. Follow-up of the infected animals was discontinued only after two consecutive negative urine qPCR results.

Blood samples were collected from the jugular or cephalic veins and drawn into BD Vacutainer tubes (BD Diagnostics, New Jersey, USA) and Venosafe^™^ tubes containing K_3_ EDTA (Terumo, Terumo Europe N.V, Leuven, Belgium) to obtain serum and whole-blood samples, respectively. Urine samples were taken aseptically by cystocentesis (males and females) or catheterization (males) during the cross-sectional study, and urine samples taken during the prospective evaluation were obtained exclusively by cystocentesis.

### Clinical evaluation

The clinical evaluation included inspection for jaundice, lymphadenopathy and hyperthermia. The shelter’s employees were instructed to report any gastrointestinal, urogenital, cardio-respiratory, nervous or behavioral disorders.

Serum biochemistry analysis was performed in a Labmax 240 device (Labtest Diagnostica, Minas Gerais, Brazil) using an Enzymatic Kinetic Method kit (Rx Series, Randox, Crumlin, UK), following the manufacturer’s specifications. The analysis included alkaline phosphatase (ALP) and alanine aminotransferase activity (ALT). Total bilirubin (TB), total protein (TP), blood urea nitrogen (BUN) and creatinine (CR) serum concentrations were also determined. Hematological analysis was performed in an ABX Micros ABC Vet (Horiba Medical, Kyoto, Japan) within four hours of sampling and included white blood cell count (WBC), red blood cell count (RBC) and platelet count (PLT). The differential WBC count was performed by optical microscopy of Rosenfeld-stained blood smears when necessary (Modified May-Grünwald). The reference intervals adopted for this study are presented in S1 Appendix.

### DNA extraction and PCR assays

The urine samples were centrifuged (10.000 x g, 25°C, 25 min), and the pellets were resuspended in 2 mL of sterile phosphate-buffered saline (PBS, pH 7.2) prior to storage at 4°C. DNA was extracted within 48 h after sample collection using the Nuclisens MiniMag Kit^®^ (BioMerieux, Inc., Durham, NC), with slight protocol modifications as follows: 1 mL of the lysis solution was used in the initial step, and the final elution was performed using 40 μL of elution buffer. DNA was extracted from the blood samples using the QIAamp DNA Mini Kit^®^ (Qiagen Inc., Valencia, CA) in accordance with the manufacturer’s instructions. All extracted DNA samples were stored at -20°C until quantitative PCR testing.

The detection of pathogenic *Leptospira* was performed using a quantitative real-time assay targeting the *lipl32* gene. The primers (forward: 5′-TCGCTGAAATRGGWGTTCGT-3′; reverse: 5′-TAAAGCCAGGACAAGCGCC-3′), probe (FAM-5′-AAAGCCAGGACAAGCGCCG-3′-MGB) and cycling conditions used had been previously validated to detect pathogenic *Leptospira* from canine urine samples [39]. Each reaction had a final volume of 25 μL, with 600 μM of each primer, 250 nM of the probe, 1x TaqMan^®^ Universal Master Mix II (Thermo Fisher Scientific Inc., Carlsbad, CA, USA), DNase free-water and 5 μL of the extracted DNA. The amplification protocol consisted of 2 min at 50°C, 10 min at 95°C and 45 cycles of amplification (95°C for 15 s and 60°C for 60 s). For absolute quantification, amplifications of serial dilutions of genomic DNA extracted from *L*. *interrogans* serovar Canicola strain Hond Utrecht IV were performed in addition to qPCR testing of the clinical samples. Cultured leptospires were quantified using a Petroff-Hausser counting chamber, and the extracted DNA was quantified in duplicate using the Qubit^®^ 2 Fluorometer (Invitrogen, Thermo Fisher Scientific Inc., Carlsbad, CA, USA) to determine the number of genomic equivalents (GE). To prepare the standard curves, the extracted DNA was standardized to an initial concentration of 1×106 GE/reaction, followed by serial 10-fold dilutions until 1×101 GE/reaction was reached. All standard-curve dilutions were tested in triplicate, and each run included a single negative control containing sterile nuclease-free water. Samples were considered positive if the cycle threshold values (Ct) from at least 2/3 replicates were higher than the Ct values from the last endpoint dilution.

To assess the quality of the DNA extraction, all clinical samples were subjected to a quantitative assay targeting the canine melanocortin-1 receptor encoding-gene (*MC1R*), as described elsewhere [40]. Clinical specimens and positive controls using DNA extracted from pure cultures of canine fibroblast cells strain A-72 (ATCC^®^ CRL-1542^™^, American Type Culture Collection, Manassas, VA, USA) were tested in duplicate and each run included a single negative control containing DNAse-free water. All qPCR runs were performed using the same equipment (Applied Biosystems^®^ 7500 Real-Time PCR System, Thermo Fisher Scientific Inc., Carlsbad, CA, USA).

To confirm the *lipl32* qPCR results, all positive samples were subjected to a partial 16S rRNA gene amplification targeting a 331bp fragment [41,42] and were thereafter sequenced to confirm the leptospiral sequence identity. Conventional PCR amplification was carried out as previously described [39]. Selected samples presenting positive yields were also subjected to a partial *secY* gene amplification, according to a previous description [41]. *L*. *interrogans* sv. Canicola genomic DNA was used as a positive control, and DNAase-free water was used as a negative control in all conventional PCR runs.

### Culturing of leptospires

To recover leptospires, 0.5 mL of each urine sample was diluted in sterile physiological solution to final concentrations of 1:10 and 1:100, and 0.5 mL of each solution was further inoculated into semi-solid Fletcher and liquid EMJH media (Difco Laboratories, Franklin Lakes, NJ, USA). The tubes were incubated at 28°C for 16 weeks and examined weekly by dark-field microscopy to confirm the presence of spirochetes.

### Microscopic agglutination test (MAT)

Antibody titration against *Leptospira* sp. was determined by MAT, following the recommended protocol (World Organization for Animal Health, 2012). The test included 22 serovars (Australis, Bratislava, Guaricura, Autumnalis, Butembo, Castellonis, Bataviae, Canicola, Whitcombi, Cynopteri, Grippotyphosa, Hebdomadis, Copenhageni, Icterohaemorrhagiae, Javanica, Panama, Pomona, Pyrogenes, Hardjo-hardjoprajitno, Shermani, Tarassovi, and Sentot) representing 18 serogroups. Endpoint titers were determined using two-fold dilutions until the last well showing 50% agglutination was recorded. The cutoff for a positive agglutination reaction was defined as a titer ≥100.

Serum samples from all dogs during the cross-sectional evaluation were also retested by MAT using leptospiral strains recovered in this study as antigens.

### Characterization of the isolated strains

#### Molecular characterization

Species identification was performed by Multilocus Sequence Typing (MLST) using seven distinct loci (*pntA*, *sucA*, *mreA*, *glmU*, *caiB*, *tpiA*, *pfkB*), as previously described [43]. Sequence types (STs) were determined from the resulting allelic profiles and compared to an established Internet database to obtain the species identification (http://pubmlst.org/leptospira).

#### Serological characterization

The strains were serogrouped using polyclonal antibodies according to previous recommendation [44]. A panel of 34 specific antisera representing 28 serogroups was used (Andamana, Australis, Autumnalis, Ballum, Bataviae, Canicola, Calledoni, Codice, Cynopteri, Djasiman, Grippotyphosa, Hebdomadis, Holland, Icterohaemorrhagiae, Javanica, Louisiana, Lyme, Manhao, Mini, Panama, Pomona, Pyrogenes, Ranarum, Sarmin, Sejroe, Seramanga, Shermani, and Tarassovi). Serogroup identification was confirmed by the highest titration against specific representative serovars included in the panel.

#### Virulence characterization

Pure cultures of each isolated strain were counted in a Petroff-Hausser chamber and 0.5 mL containing 10^8^ leptospires was intraperitoneally inoculated into thirty-day-old male hamsters (*Mesocricetus auratus*) and guinea pigs (*Cavia porcellus*) to determine if the isolates would produce clinical signs of leptospirosis [45]. The animals were purchased at Anilab Animais de Laboratório Criação e comércio, Paulínia, SP, and were bred strictly for research purposes. The hamsters were kept in individual 30 X 20 X 12 cm polypropylene cages lined with wood shavings; free fresh water and food was daily supplied and the animals were housed at an isolated laboratory animal house with controlled temperature. The guinea pigs were kept in individual 80 X 80 X 60 cm steel cages and were kept under the same conditions described for the hamsters.

After inoculation, the animals were daily monitored for signs of acute leptospiral infection, including prostration, ruff hair coat, jaundice, external hemorrhage and dehydration. In order to meet the pre-established humane endpoints criteria and to minimize animal suffering and distress, the animals were promptly euthanized after showing two or more clinical signs, and asymptomatic animals were euthanized 21 days post-infection. The kidneys were aseptically removed, macerated, resuspended and inoculated in liquid EMJH medium for the re-isolation of leptospires.

The DU114 strain was inoculated into two hamsters and the DU92 strain was inoculated into eight hamsters (one initial inoculation and seven further *in vivo* passages); leptospires recovered from the last DU92 *in vivo* passage were also inoculated into four guinea pigs to evaluate the strain virulence in a different animal model and to evaluate the possibility of pathogenicity activation of this particular strain.

Kidney tissues from the animal models presenting no signs of infection were tested by the 16S rRNA PCR, and serum samples were tested by MAT.

### Sequencing analysis

The MLST, 16S rRNA and *secY* amplicons were separated on a 2% agarose gel stained with SYBRSafe DNA Gel Stain (Invitrogen, Carlsbad, CA, EUA) for further observation using UV transillumination. DNA fragments were purified using the Wizard^®^ SV gel and the PCR Clean-up System (Promega Corporation, Madison, EUA). DNA sequencing was carried out on an ABI 7500 Genetic Analyzer (Applied Biosystems Foster City, CA, USA) using the Big Dye Terminator 3.1 Cycle Sequencing Kit (Life Technologies Corporation, Carlsbad, CA, USA) according to the manufacturer’s instructions.

### Statistical and phylogenetic analysis

The comparative MAT and quantitative PCR results were analyzed using Sigma Stat for Windows version 3.0 (SPSS, Inc). Pearson’s chi-square test was performed to evaluate associations in the qualitative data, and the Mann-Whitney test was used for quantitative data; p values lower than 0.05 were considered statistically significant.

**Fig 1:**
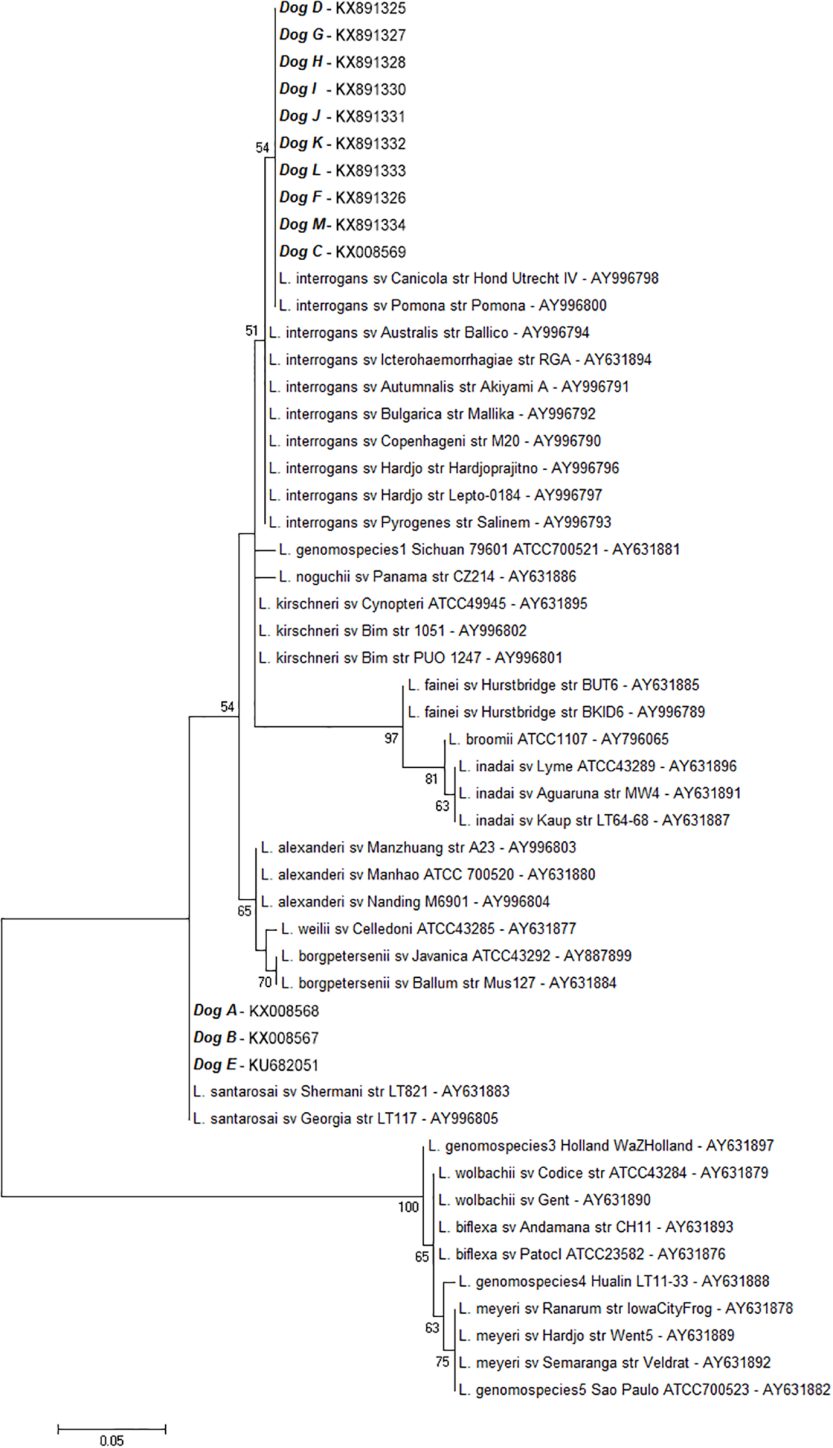
*Leptospira* species confirmation based on the 16S rRNA phylogenetic analysis using the maximum-likelihood method with nearest neighbour interchanges. The bootstrap values presented at corresponding branches were evaluated using 1000 replicates. All GenBank accession numbers are shown next to each recovered sequence.

For the 16S rRNA and *secY* phylogenetic analysis, the consensus sequences were aligned with GenBank reference sequences and phylogenetic trees were constructed using Mega 5.10 software using the maximum-likelihood method with nearest neighbor interchanges; 1000 bootstrap replicates were used for branch support statistical inference. For the MLST analysis, Bionumerics 7.6 (Applied Maths NV, Sint-Martens-Latem, Belgium) was used to compare the concatenated loci to *Leptospira* sequence types (STs) available on the PubMLST database using the maximum-likelihood method. All the obtained sequences were submitted to the PubMLST and GenBank repositories (accession numbers are shown in Figs 1 and 4).

### Ethical considerations and drug protocols

All procedures involving animals were approved by the Ethical Committee of the School of Veterinary Medicine of the University of São Paulo (protocol 2706/2012). All chronically infected animals were properly treated using three different protocols, following previous recommendations [46,47]. The protocols included (a) a single subcutaneous dose of streptomycin 25 mg/kg, (b) oral doxycycline 4 mg/kg for 14 days, or (c) a single subcutaneous dose of ceftriaxone 30 mg/kg. The antimicrobial therapy was instituted if any of the following conditions were observed: (I) someone showing interest in adopting one of the infected dogs, (II) after the confirmation of leptospiral recovery in culture media, or (III) if the infected animal exhibited any underlying condition that required antimicrobial therapy. The euthanasia procedures conducted in the animal models were in strict accordance with the recommendations in the CONCEA (National Council for Control of Animal Experimentation), and consisted of intraperitoneal administration of xylasine/ketamine and isofluran, followed by the use of a CO_2_ chamber. The procedure was approved by the Ethical Committee of the School of Veterinary Medicine of the University of São Paulo (protocol 1761220216), and all efforts were made to minimize animal suffering.

## Results

### Cross-sectional evaluation

Out of the 24 dogs kept at the Mogi das Cruzes shelter, only one (4.1%) had leptospiral DNA in its urine (dog A, Table 1). No serum titers against *Leptospira* were detected in the sheltered dogs and none of the dogs had been immunized against leptospirosis. All dogs had been kept at the shelter for at least six months.

**Table 1:** qPCR and MAT results, estimated bacterial load, major laboratorial and clinical findings and registry data from all dogs exhibiting urinary shedding of leptospires.

Dog ID	Leptospires/mL urine	Interval between check-in and sample collection	Interval between immunization and sample collection	Major laboratory and clinical findings	MAT results									
					PO	BU	CAS	IC	PY	BRA	AUT	HA	WO	GRI
**A**	40	> 6 months	Up to 6 months	Vaginal neoplasia	-	-	-	-	-	-	-	-	-	-
**B**	76	Stray dog	Unknown	Unremarkable	-	-	-	-	-	-	-	400	400	200
**C**	160	Stray dog	Up to 6 months	Unremarkable	-	-	-	-	-	-	-	-	-	800
**D**	682,545	2 months	1 month	Unremarkable	800	-	-	800	800	100	100	-	-	-
**E**	44	> 6 months	Up to 6 months	Unremarkable	-	-	-	-	-	-	-	-	-	-
**F**	137,183	1 hour	Unknown	*See* S3 Appendix	-	-	-	-	200	-	-	-	-	-
**G**	30	>6 months	4 months	Unremarkable	-	-	-	-	-	-	100	-	-	-
**H**	118	>6 months	Up to 6 months	Unremarkable	-	-	-	-	-	-	-	-	-	-
**I**	6,348	>6 months	Up to 6 months	Unremarkable	-	-	-	-	-	-	-	-	-	200
**J**	28	>6 months	Up to 6 months	Unremarkable	-	-	100	100	200	-	100	-	-	-
**K**	30	>6 months	Up to 6 months	Unremarkable	-	-	-	-	-	-	-	-	-	-
**L**	273	>6 months	1 month	Unremarkable	400	400	400	-	-	-	-	-	-	-
**M**	2,046	1 hour	Unknown	Unremarkable	-	-	-	-	-	-	-	-	-	-

PO: sv. Pomona; BU: sv. Butembo; CAS: sv. Castellonis; IC: sv. Icterohaemorrhagiae; PY: Pyrogenes; BRA: sv. Bratislava; AUT: sv. Autumnalis; HA: sv. Hardjo; WO: sv. Wolffi; GRI: sv. Grippotyphosa

Out of the seven stray dogs living on the USP campus, two (28.5%) exhibited positive qPCR results in urine samples (dogs B and C, Table 1). Six out of the seven dogs (including dogs B and C) had anti-*Leptospira* serum titers (ranging from 100 to 800), and most dogs had antibodies against serogroups Grippotyphosa (n = 4), Autumnalis (n = 2), Pomona (n = 1), Icterohaemorrhagiae (n = 1) and Canicola (n = 1). Only one dog (Dog B) exhibited serum titers (400) against Sejroe serogroup. All MAT-positive dogs, except for dog B, had been immunized against leptospirosis (Recombitec^™^ C6/CV, Merial Inc., Georgia, USA) within 6 months prior to the sample collection.

Out of the 92 dogs kept at the USP shelter, 10 (10.87%) had leptospiral DNA in their urine (dogs D to M, Table 1). Serum antibodies against *Leptospira* sp. were detected in 47 dogs (51%, including dogs D, F, G, J and L, Table 1), with titers ranging from 100 to 12,800. Most dogs had antibodies against the serogroups Autumnalis (n = 43), Icterohaemorrhagiae (n = 34), Pomona (n = 20) and Pyrogenes (n = 14). Less common serogroups found were Canicola, Wolffi and Shermani (n = 1 for each serogroup). Of the 92 dogs, 36 had been immunized against leptospirosis less than six months before the samples were taken, while 33 dogs had been immunized more than six months before sample collection. The immunization status of 23 dogs could not be determined, namely the 11 dogs that had been recently admitted to the facility and the 12 dogs without immunization records. Table 2 shows the highest titers found in MAT-positive dogs from the USP shelter according to their immunization status. There was a significant difference in MAT results between the immunization categories (p = 0.0009); the presence of antibodies was more frequent in recently vaccinated dogs when compared to dogs immunized more than six months before sample collection or dogs with unknown immunization status (p = 0.0081 and p = 0.0021, respectively). There was no significant difference between dogs with no immunization records and dogs vaccinated more than six months before sample collection (p>0.05).

**Table 2:** MAT results and maximum serum antibody titers against *Leptospira* sp. found in the 92 dogs kept in the USP shelter, grouped according to their immunization status.

Interval between immunization and sample collection	MAT Negative	MAT Positive (≥100)	TOTAL	Maximum MAT titration found in MAT-positive dogs				
				100	200	400	800	≥1,600
**<6 months**	9	27	**36**	4	9	7	1	6
**>6 months**	20	13	**33**	3	7	3	0	0
**Unknown**	16	7	**23**	0	3	2	2	0
**TOTAL**	**45**	**47**	**92**	**7**	**19**	**12**	**3**	**6**

The median volumes of all qPCR-negative urine samples (9.5 mL) were not significantly greater than those of PCR-positive urine samples (9.2 mL), and the number of leptospires found in qPCR-positive dogs ranged from 30 to 682,545 leptospires/mL (Table 1). No amplification of leptospiral DNA in blood samples or clinical abnormalities related to canine leptospirosis were observed among the dogs included in this study, except for dog F, which presented slightly pale mucous membranes and increased BUN/CR levels at the clinical examination. All qPCR-negative urine and blood samples taken from the dogs of the three populations tested positive for the *MC1R* assay, and the standard curves tested positive in all qPCR runs, indicating adequate DNA extraction and amplification procedures. The number of leptospires/ml urine, MAT results, laboratory findings and relevant information regarding the registration and immunization status of all qPCR-positive dogs are presented in Table 1. The remaining data from all dogs included in the cross-sectional study are available in S2 Appendix.

The 16S rRNA phylogenetic analysis (Fig 1) showed that ten of the recovered sequences were identified as *L*. *interrogans* (dogs C, D, F, G, H, I, J, K, L and M), presenting high similarity (> 99%) with *L*. *interrogans* serovar Canicola strain Hond Utrecht IV (AY996798) and *L*. *interrogans* serovar Pomona strain Pomona (AY996800). The leptospiral DNA found in urine samples taken from three dogs (dogs A, B and E) presented sequences with > 98% similarity with *L*. *santarosai* serovar Shermani strain LT821 (AY631883) and *L*. *santarosai* serovar Georgia strain LT117 (AY996805).

### Prospective evaluations

Three leptospiruric dogs could not be reevaluated (Table 3): dog A was euthanized two days after the first sample collection due to clinical complications attributed to a vaginal neoplasia, dog B could not be located again, and dog D was adopted after the first evaluation, hampering its inclusion in the prospective evaluation. The remaining 10 leptospiruric dogs were regularly reevaluated (Table 3), with a mean interval of 11,54 days between evaluations (SD 10.97). Seven leptospiruric dogs presented intermittent urinary shedding of leptospires, and reevaluations were discontinued after exhibiting two negative and consecutive urinary qPCR results. No seroconversion, positive blood qPCR results or clinical/laboratorial abnormalities were observed in these seven dogs, and it was not possible to recover leptospires in culture media from these animals.

**Table 3:** Urinary qPCR results found during the prospective evaluation of the leptospiruric dogs.

Dog ID	Evaluation and urine qPCR results															
	1^st^	2^nd^	3^rd^	4^th^	5^th^	6^th^	7^th^	8^th^	9^th^	10^th^	11^th^	12^th^	13^th^	14^th^	15^th^	16^th^
**A**	+	E														
**B**	+	NF														
**C**	+	+	+	+	-*	-*										
**D**	+	A														
**E**	+	+	+	+	+	+	+	+	+	+	+	+	+	+	-*	-*
**F**	+	+	+	+	+	+	+	+	+	+	-*	-*				
**G**	+	-	-													
**H**	+	-	-													
**I**	+	-	-													
**J**	+	-	-													
**K**	+	+	-	-												
**L**	+	-	-													
**M**	+	-	-													

*: Post-treatment evaluation; E: Euthanasia; NF: Not found; A: Adopted.

Three dogs (dogs C, E and F) presented persistent urinary shedding of leptospires, with positive qPCR results on several occasions (Table 3). Dog C (male, adult, recently vaccinated) was examined six times throughout a 22-week period, with a mean interval of 30.68 days between evaluations (SD 26.01). It was not possible to recover leptospires in culture media, and leptospiruria was interrupted only after the dog was treated with a single subcutaneous dose of ceftriaxone (Rocefin^®^, Roche, Rio de Janeiro, RJ, Brazil). Two urine qPCR evaluations performed seven and 14 days after the administration of the drug showed negative results, indicating possible therapeutic success. The dog did not exhibit seroconversion or any relevant clinical/laboratorial abnormalities, and no amplification of leptospiral DNA was observed in blood samples throughout the evaluation period.

**Fig 2:**
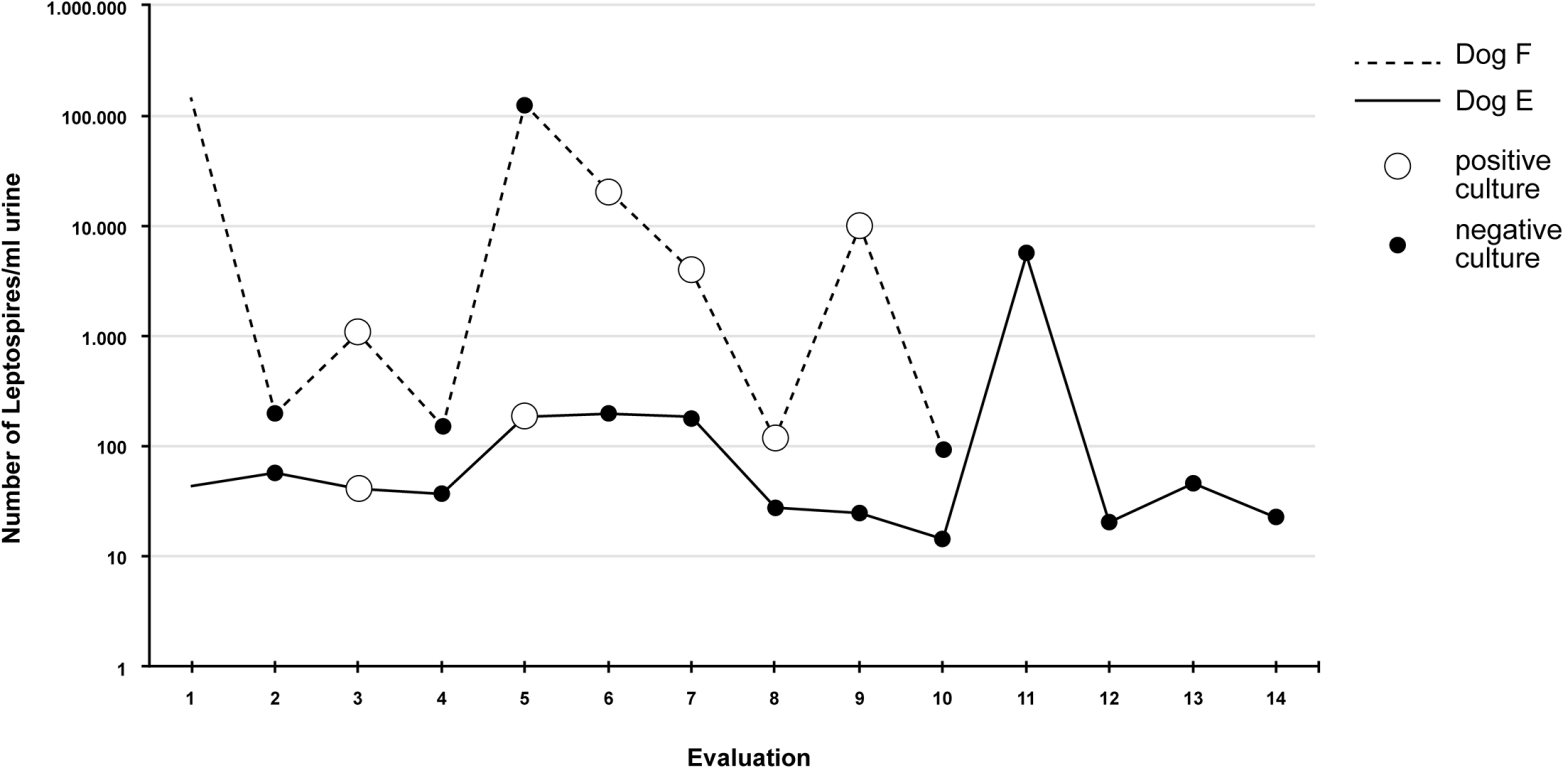
Number of leptospires/mL urine and isolation results from the evaluations performed in dogs E and F.

Dog E (male, adult, unvaccinated) was evaluated on 16 occasions throughout a 18-week period, with a mean interval of 8.47 days between evaluations (SD 2.92). The urinary qPCR tested positive for all samples, and leptospires were recovered on two different occasions. Fig 2 shows the number of leptospires/mL detected in each evaluation, along with the results of the isolation attempts. After the 14^th^ evaluation, the dog was treated with streptomycin (Estreptomax^®^, Ourofino, Cravinhos, SP, Brazil), and no leptospiral DNA was detected in the urine samples collected seven and 14 days post-treatment. No antibody titers against *Leptospira* were detected during the evaluations. The dog did not present any relevant clinical/laboratorial abnormalities throughout the evaluation period, and no amplification of leptospiral DNA was observed in the blood samples.

Dog F (male, adult, unknown immunization status) was evaluated on 12 occasions throughout a 12-week period, with a mean interval of 7.73 days between evaluations (SD 3.49). The dog was captured on the University campus and had samples taken immediately after admittance at the USP shelter. Dog F presented slightly pale mucous membranes during the first evaluation and was immunized against leptospirosis immediately after admission. Leptospiral DNA was detected in urine samples from all revaluations. Dog F presented significantly more leptospires in urine samples than dog E throughout the evaluations (p = 0.002), and leptospires were recovered on five different occasions (Fig 2).

The laboratorial evaluation of dog F revealed blood smear visualization of *Anaplasma* sp, a low platelet count, low hematocrit and high BUN/CR levels on more than one occasion (S3 Appendix). After the 10^th^ evaluation, the dog was treated with doxycycline (Doxitrat^®^, Agener, Embu, SP, Brazil). No leptospiral DNA was detected in urine samples taken seven and 14 days after the antibiotic intervention; no further clinical signs of canine anaplasmosis were observed.

All urine and blood samples taken during the revaluations that contained no pathogenic *Leptospira* tested positive for the *MC1R* gene. All data obtained during the prospective evaluation can be visualized in the S2 Appendix.

### Characterization of the isolated strains

One isolate recovered from each dog was selected for further molecular, serological and virulence characterization. The strains isolated from dog E and dog F were registered as DU92 and DU114, respectively.

**Fig 3:**
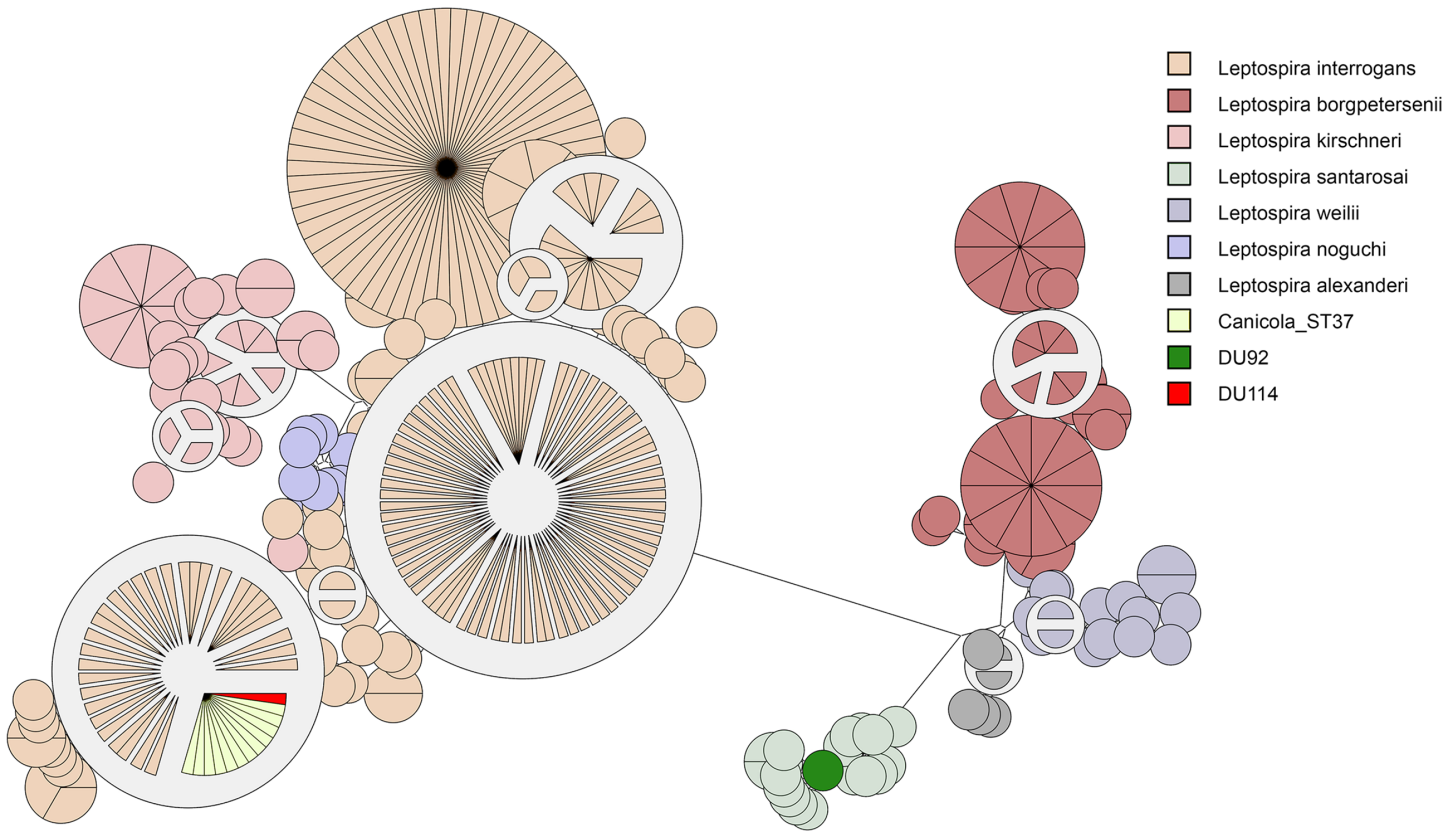
MLST analysis of the DU92 and DU114 strains. The maximum-likelihood tree was based on the concatenated sequences of the seven loci for the 229 available *Leptospira* STs. The strain DU92 was registered on the PubMLST database under ID number 525.

The characterization of the DU92 strain revealed a strong and specific reaction against serogroup Sejroe and the MLST analysis revealed a new sequence type (ST218 –Fig 3), characterizing the strain as *L*. *santarosai*, as previously described by our group [28]. The hamster inoculated with the DU92 strain did not present any clinical signs of acute leptospirosis. However, leptospires were recovered in culture media from kidney tissues 21 days post-inoculation. Seven *in vivo* passages were performed using the DU92 strain without producing clinical signs of infection. Similarly, none of the four guinea pigs presented clinical signs of leptospirosis. It was not possible to recover leptospires from the guinea pig kidney samples 21 days after experimental infection, and none of the samples exhibited leptospiral 16S rRNA amplification. However, MAT titers exclusively against serogroup Sejroe were detected in 5/8 animals (2/4 hamsters and 3/4 guinea pigs), ranging from 100 to 400 against serogroup Sejroe.

The MLST analysis of the DU114 strain revealed a ST37 profile, which characterizes *L*. *interrogans* serovar Canicola, according to a previous described protocol [43]; Fig 3 shows the clustering of the DU114 isolate with *L*. *interrogans* serovar Canicola STs. Serogrouping also revealed a strong and specific titration against serogroup Canicola (12,800 for serovar Canicola, 3,200 for Icterohaemorrhagiae, and 800 for serovars Castellonis, Mini and Pyrogenes). The strain was virulent in the hamster model. Both hamsters used for virulence characterization developed two or more signs established as humane endpoint criteria within five days after the inoculation and were promptly euthanized. Macroscopic alterations after euthanasia included epistaxis, generalized petechial stains, pulmonary/liver congestion and pulmonary hemorrhage. Leptospires were successfully recovered from kidney and liver tissues from both hamsters after euthanasia.

### *secY* phylogenetic analysis

**Fig 4:**
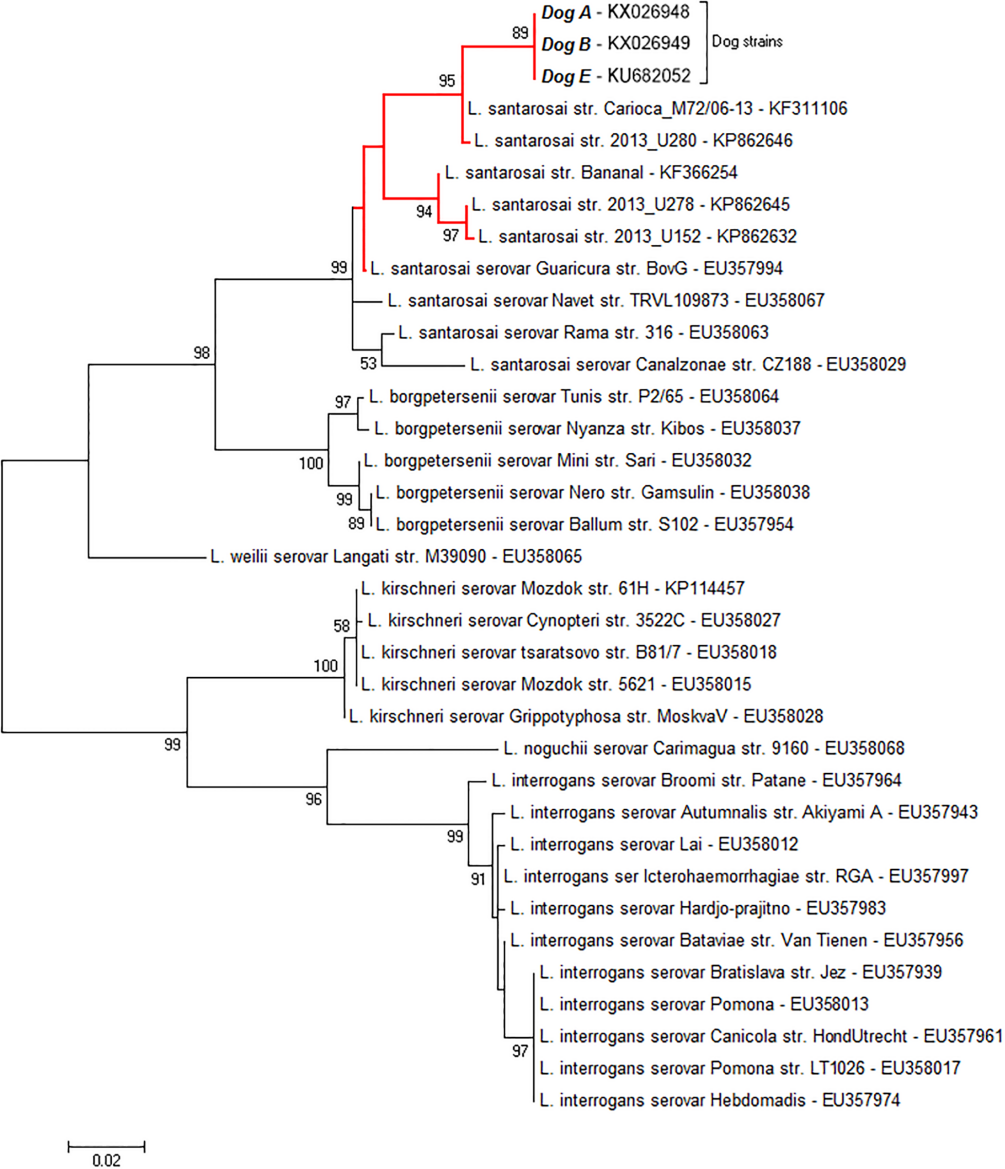
Maximum-likelihood tree constructed with the Tamura-Nei model and nearest neighbor interchanges using 1000 bootstrap replicates. The red branches feature sequences of Brazilian *L*. *santarosai* strains used for the comparative analysis. All GenBank accession numbers are shown next to each recovered sequence.

The *secY* phylogenetic analysis was performed using DNA exclusively from dogs exhibiting urinary shedding of *L*. *santarosai*. The analysis included the DU92 strain, as well as other publicly available sequences of *L*. *santarosai* recovered from different host species. It revealed a high sequence identity between sequences recovered from dogs A, B and E (S4 Appendix), showing also that these sequences clustered separately from the *secY* gene sequences obtained from other hosts (Fig 4).

### MAT results using the local isolates

Serum samples collected from all dogs during the cross-sectional evaluation (123 samples) were retested by MAT using the DU92 and DU114 strains as antigens. No increased seroreactivity was detected in the samples tested.

## Discussion

Dogs exhibiting asymptomatic urinary shedding of pathogenic *Leptospira* were successfully identified in all populations studied, and the prospective evaluation of the infected dogs has confirmed the presence of chronic carriers among these populations, allowing the culturing and characterization of circulating leptospiral strains. Even though longitudinally designed studies may provide tools to effectively characterize chronically infected dogs rather than merely identifying the transient urinary shedding of leptospires, few studies have investigated canine leptospirosis using a prospective approach [48,49]. To the best of our knowledge, the present study stands as the first prospective evaluation using multiple diagnostic methods for detecting persistent and asymptomatic leptospiral infection in dogs.

The serological findings revealed that a large proportion of the dogs sampled during the cross-sectional evaluation had anti-*Leptospira* antibodies detectable by MAT. The majority of seroreactive dogs, however, had been recently vaccinated, and the highest titers (≥1,600) were detected exclusively in serum from recently immunized dogs. Although post-vaccinal titers are usually low and could persist for only a few months [50], high titration during the early post-vaccination period has been reported [51]. The serological pattern found indicates that most MAT-positive reactions were associated with vaccination. Moreover, most dogs reacted against representative serovars from the serogroups Icterohaemorrhagiae, Autumnalis and Pomona, which are frequently included in vaccine compositions and commonly associated with post-vaccinal cross reactions [47,52]. Only a smaller proportion of dogs had no history of recent vaccination, and MAT titers found in these animals possibly indicate recent exposure to leptospires. It is noteworthy that some of these dogs had been kept for more than six months in the local shelters, indicating the circulation of the pathogen inside both facilities.

Although a more thoughtful interpretation of MAT results has provided some interesting insights into epidemiological features surrounding the populations studied, only seven out of 13 leptospiruric dogs presented concomitant MAT titers, three of which had been recently vaccinated, thus confirming that MAT is not a suitable technique for the identification of asymptomatic leptospiral infection [47].

The proportion of leptospiruric dogs was similar to those found in stray and sheltered dog populations from different locations [12,15,25], and the 16S rRNA sequence analysis revealed that most dogs exhibited urinary shedding of *L*. *interrogans*. Interestingly, urinary shedding of *L*. *santarosai* was detected in dogs from the three populations studied. The phylogenetic analysis of the *secY* gene revealed that the *L*. *santarosai* strains infecting those dogs were very similar to each other, forming a separate cluster when compared to *L*. *santarosai* recovered from other mammalian species. These results suggest a possible genetic distinction between *L*. *santarosai* maintained by dogs from those maintained by other animal hosts. Furthermore, one of the dogs presenting *L*. *santarosai* infection was successfully characterized as a chronic carrier, as previously suggested [28], evidencing that this particular strain was fully adapted to persistently colonize the dog’s renal tubules.

Asymptomatic renal carriage of *L*. *santarosai* is typically observed in wild animals, such as raccoons and wild rodents [53]. It has also been recovered from different domestic species, such as goats [54], cattle [55] and water buffaloes [56]. Recent whole-genome sequencing of *L*. *santarosai* revealed genomic regions encoding transposases and hypothetical proteins that may enhance fitness, possibly allowing the successful renal colonization of such a variety of mammalian hosts [57].

The identification of dogs exhibiting asymptomatic *L*. *santarosai* infection found in this study may contribute to dispelling the concept that the canine species acts as exclusive carriers of *L*. *interrogans* strains. In this context, different *Leptospira* species have also been recovered from asymptomatic dogs, such as *L*. *kirschneri*, *L*. *wolffi*, *L*. *weilli* and *L*. *borgpetersenii* [14,21,22,58–60]. Asymptomatic infection caused by different *L*. *interrogans* serovars/serogroups, such as Copenhageni/Icterohaemorrhagiae [15,17,23,49,61,62], Pomona [15,63,64], Tarassovi [64], Sejroe [65,66] and Hebdomadis [67] have also been reported, further revealing that the classical association of particular serovars with specific maintenance hosts may not be absolute. These observations, along with our findings, highlight that the transmission from dogs cannot be assessed exclusively by isolating Canicola strains from accidental hosts, and that renal carriage of such a variety of pathogenic *Leptospira* cannot be overlooked by local Public Health authorities. Acute infection caused by *L*. *santarosai* was recently reported in dogs [68], and *L*. *santarosai* has been increasingly identified as the causative agent of severe cases of human leptospirosis [69–71]. Our results suggest a possible and unexpected role of dogs in the chain of transmission of this leptospiral species in urban environments, and asymptomatic carriage of *L*. *santarosai* in dogs demands further investigation.

The DU92 strain is the first reported *L*. *santarosai* strain ever isolated from a dog, as previously described by our group [28]. In the present study, however, the usage of the DU92 strain as an antigen to retest serum samples has failed to increase seroreactivity against serogroup Sejroe, and the strain virulence could not be demonstrated by inoculation in the animal models, with both hamsters and guinea pigs failing to demonstrate signs of leptospirosis. Still, serum titers against serogroup Sejroe could be detected in both species used for virulence testing, showing that the strain could produce a specific immune response after experimental inoculation. Curiously, serum titers against serogroup Sejroe were also detected in one of the three dogs infected by *L*. *santarosai* (dog B), possibly indicating the circulation of the DU92 strain around the campus of the University of São Paulo. Symptomatic and asymptomatic infection caused by serogroup Sejroe was already reported in dogs [65,66], revealing the possibility of horizontal transmission of this serogroup among dogs kept under shelter conditions [66].

Among the 10 animals with infection caused by *L*. *Interrogans*, two dogs could be characterized as chronic carriers. One of them (dog F) presented prolonged urinary shedding of leptospires confirmed by qPCR, irrespective of its immunization status, allowing the recovery of leptospires on multiple occasions (strain DU114). The strain was characterized as *L*. *interrogans* serogroup Canicola, which was demonstrated to be pathogenic in the hamster model. Canicola strains have been consistently recovered from dogs in Brazil [72], and human cases of leptospirosis are frequently attributed to this serovar [17,18,72,73]. The usage of the DU114 strain as an antigen to retest serum samples failed to increase seroreactivity against serovar Canicola. It has been suggested that the inclusion of local strains as antigens in MAT panels may increase the serodiagnostic sensitivity for leptospirosis [74]; however, conflicting results have also been reported [75].

Dog F presented a moderate increase in serum BUN/CR levels and hematological abnormalities combined with urinary shedding of leptospires during the first evaluation. Acute leptospirosis was suspected; however, the BUN/CR levels decreased rapidly during the following evaluations, and no leptospiral DNA could be amplified from blood samples taken during any evaluation. Moreover, no marked clinical abnormalities were observed during the reevaluations, and the serum anti-*Leptospira* titers found during the follow-up examinations were probably attributed to the immunization performed immediately after the dog’s admission at the local shelter. The persistence of relatively low platelet and hematocrit values and the severely low platelet count observed during the 9^th^ evaluation led our group to investigate possible alternative causes for the hematological disturbances. The retrospective visualization of blood smears revealed *Anaplasma* sp. morulae infecting platelets in blood samples taken on different occasions, and the SNAP 4Dx^®^ test (Idexx, Westbrook, USA) confirmed *Anaplasma platys* infection. These findings suggest that the hematological and clinical abnormalities found in dog F were possibly associated with *A*. *platys* infection. The dog was promptly treated with doxycycline after the co-infection was confirmed. The use of doxycycline is recommended to treat several bacterial infections in dogs, including *Anaplasma sp*. and *Leptospira* sp. infections [47].

The consecutive positive qPCR results evidenced the successful renal colonization by both DU92 and DU114 strains, and significantly different mean quantities of leptospiral DNA were detected in urine samples from each infected dog. Dog F presented significantly higher numbers of urinary leptospires than Dog E, which was infected by *L*. *santarosai*. Differences in leptospiral loads shed by asymptomatically infected dogs have been recently reported [76], but no correlation between the infecting species and the mean quantity of bacteria found in urine has been established in the literature. Our results indicate that the leptospiral load shed in the urine of infected reservoirs may be influenced by the infecting strain, albeit no profile or pattern of bacterial shedding can be further defined based exclusively on these data.

Although the Mogi das Cruzes shelter did not have any implemented vaccination protocol, the low proportion of seroreactive and leptospiruric dogs apparently indicates that proper hygiene management protocols could be partially effective at preventing leptospiral infection. Conversely, the USP shelter presented several environmental conditions that promote leptospiral transmission, and the adoption of a non-standardized vaccination protocol apparently could not prevent leptospiral infection among the housed dogs. There was a low overall turnover of animals at the USP shelter, and it became obvious that adoption could not balance the admission flow on a long-term basis, leading to increasingly overpopulated enclosures. Most unsanitary conditions were caused or aggravated by inadequate allocation of dogs. Such conditions are associated with higher prevalence of leptospiral infection among sheltered dogs and may represent serious occupational risks to kennel workers [29,35,77]. Of the ten dogs identified as urinary shedders in the USP shelter, two of them had been recently admitted to the facility. Leptospires in the urine sample taken from dog D were detected only after the adoption procedure, and it was not possible to contact the adopters for proper treatment. Both of these observations indicate that leptospiruric dogs can be inadvertently admitted and adopted at dog shelters, potentially increasing the risks of dog-to-human transmission by bringing pathogenic strains closer to adopters and caretakers [15,35,37].

Although vaccination can potentially decrease the risk of infection [78], its use as a single strategy to prevent leptospiral infection can be only partially effective in shelter environments, as our results have shown. Simultaneous control strategies should be implemented to prevent canine leptospirosis in these locations, such as highly standardized hygiene protocols, rodent infestation control, rigorous vaccination protocols and proper management of asymptotically infected dogs. Identification of leptospiruric dogs, however, requires specific laboratorial techniques (e.g. PCR, culture, dark-field microscopy), which are often not available in a shelter on a daily basis. As an alternative, asymptomatic infection can be prophylactically treated with appropriate antibiotics [47]. All antibiotic protocols used in this study successfully interrupted the urinary shedding of leptospires. The use of streptomycin, despite being effective and practical in a shelter routine, is not recommended in several locations for its potential nephrotoxicity [79]. Conversely, doxycycline is currently considered the elective drug to prevent leptospiral shedding [47]. Its use, however, requires a 14-day oral treatment, limiting its application in quarantine and adoption protocols at shelters with high turnover rates. Ceftriaxone, which presents good antibacterial sensitivity against leptospires [46] and has been recommended to replace penicillin in the treatment of acute leptospirosis in humans [80], was also able to interrupt urinary shedding of leptospires in the present study. Despite its apparent success, the use of ceftriaxone in chronically infected dogs should be further investigated. Our findings highlight that efforts should be addressed to evaluate alternative drug protocols suitable for a shelter routine, allowing secure, practical and efficient use of antibiotics to prevent the adoption and admission of asymptomatically infected animals.

## Conclusions

The use of multiple diagnostic methods enabled the successful identification of chronic carriers of pathogenic *Leptospira* among the sheltered and stray dog populations studied, allowing further identification of the infecting strains. Proper characterization of leptospiral isolates is instrumental for the implementation of strategic prevention policies by local Pubic Health authorities, and our results highlight the contribution of dogs to the chain of transmission of *L*. *santarosai* in urban environments. This study also showed that leptospiruric dogs can be inadvertently admitted and adopted in dog shelters, potentially increasing the risks of occupational and zoonotic transmission, and that the implementation of prophylactic control can be beneficial to overcome diagnostic difficulties related to the identification of asymptomatically infected individuals.

## Supporting information

S1 Appendix Reference ranges adopted for this study. (DOCX)

S2 Appendix Raw data regarding clinical and laboratorial findings, PCR, MAT results and registry information of all dogs included in the study. (XLSX)

S3 Appendix Relevant laboratorial findings and MAT results found in samples taken during the evaluations of dog F. (DOCX)

S4 Appendix Sequence identity matrix (%) of the *secY* sequences recovered from dogs infected by *L*. *santarosai*. (DOCX)
