# Supplementary material for: Prospective study of canine leptospirosis in shelter and stray dog populations: Identification of chronic carriers and different Leptospira species infecting dogs
Source: PLoS One. 2018 Jul 11;13(7):e0200384. doi: 10.1371/journal.pone.0200384 (PMC6040711; doi:10.1371/journal.pone.0200384)
Supplement: S1 Appendix — (DOCX) [file pone.0200384.s001.docx]

**S1 Appendix:** Reference ranges adopted for this study.

| Parameters | Units | Reference ranges | |
| --- | --- | --- | --- |
| ERITROGRAM |  | Minimum | Maximum |
| Erythrocytes | x10^6^/µL | 5.0 | 8.0 |
| Haemoglobin | g/dL | 12.0 | 18.0 |
| Haematocrit | % | 37 | 57 |
| *MCV | fL | 60.0 | 77.0 |
| ** MCH | pg | 22.0 | 27.0 |
| *** MCHC | % | 31.0 | 36.0 |
| LEUCOGRAM |  |  |  |
| Total Leucocytes | /µL | 6.000 | 15.000 |
| Neutrophils | /µL | 3.000 | 12.100 |
| Lymphocytes | /µL | 1.500 | 5.000 |
| Monocytes | /µL | 0 | 800 |
| Eosinophil | /µL | 0 | 1.300 |
| Basophils | /µL | 0 | 140 |
| Platelet count | x10³/µL | 200 | 600 |
| SERUM BIOCHEMISTRY |  |  |  |
| Plasmatic protein | g/dL | 5.3 | 7.6 |
| Albumin | g/dL | 2.3 | 3.8 |
| ALT | U/L | 10 | 88 |
| Alkaline phosphatase | U/L | 20 | 150 |
| BUN | mg/dL | 20 | 80 |
| Creatinine | g/dL | 0.7 | 1.4 |

*Mean corpuscular volume; ** Mean corpuscular hemoglobin; *** Mean corpuscular hemoglobin concentration.
