# Supplementary material for: Prospective study of canine leptospirosis in shelter and stray dog populations: Identification of chronic carriers and different Leptospira species infecting dogs
Source: PLoS One. 2018 Jul 11;13(7):e0200384. doi: 10.1371/journal.pone.0200384 (PMC6040711; doi:10.1371/journal.pone.0200384)
Supplement: S3 Appendix — (DOCX) [file pone.0200384.s003.docx]

**S2 Appendix:** Relevant laboratorial findings and MAT results found in samples taken during dog F evaluations

| Dog F evaluation | Laboratorial findings | | | | | MAT | | | | | | | | |
| --- | --- | --- | --- | --- | --- | --- | --- | --- | --- | --- | --- | --- | --- | --- |
|  | BUN  mg/dL | CR  mg/dL | PLT  (${10}^{3}$/$\mathrm{mm}^{3}$) | Blood smear findings | SNAP 4Dx® test | PY | BRA | CAS | IC | PO | GRI | CA | BU | AUT |
| 1 | 185,7 | 2,28 | 55 | *A. platys* (+++), reactive lymphocytes (+++) | negative | 200 | - | - | - | - | - | - | - | - |
| 2 | 122,5 | 1,38 | 134 | reactive lymphocytes (+++), reactive monocytes (++) |  | 100 | - | - | - | - | - | - | - | - |
| 3 | WRR | WRR | 238 | reactive lymphocytes (+++), reactive monocytes (++) |  | 800 | 400 | - | - | - | - | - | - | - |
| 4 | 90 | 1,43 | 173 | reactive lymphocytes (++), reactive monocytes (+) |  | 200 | 400 | - | - | - | - | - | - | - |
| 5 | WRR | WRR | 190 | *A. platys* (+++), reactive lymphocytes (+) | positive for A. platys | 200 | 200 | - | 100 | 100 | - | - | 100 | - |
| 6 | WRR | WRR | 140 | reactive lymphocytes (+++), reactive monocytes (+) |  | - | 200 | - | - | 200 | - | - | - | - |
| 7 | WRR | WRR | 152 | reactive monocytes (+) |  | - | - | 200 | 100 | - | 100 | 400 | - | 100 |
| 8 | WRR | WRR | 100 |  |  | - | - | 200 | 100 | - | 100 | 400 | - | 100 |
| 9 | WRR | WRR | 36 |  |  | - | - | 400 | 200 | - | - | 200 | - | 200 |
| 10 | WRR | WRR | 224 |  |  | - | - | 200 | - | - | - | 400 | - | 100 |

WRR: Within reference range; PY: Pyrogenes sv.; BRA: Bratislava sv.; CAS: Castelonis sv.; IC: Icterohaemorrhagiae sv.; PO: Pomona sv.; GRI: Grippothyphosa sv.; CA: Canicola sv.; BU: Butembo sv.; AUT: Autumnalis sv..
