# Supplementary material for: Prospective study of canine leptospirosis in shelter and stray dog populations: Identification of chronic carriers and different Leptospira species infecting dogs
Source: PLoS One. 2018 Jul 11;13(7):e0200384. doi: 10.1371/journal.pone.0200384 (PMC6040711; doi:10.1371/journal.pone.0200384)
Supplement: S4 Appendix — (DOCX) [file pone.0200384.s004.docx]

**S3 Appendix:** sequence identity matrix (%) of the *secY* sequences recovered from dogs infected by *L. santarosai.*

| *L. santarosai* serovar Navet str. TRVL109873 - EU358067 | ID |  |  |  |  |  |  |  |  |  |  |  |
| --- | --- | --- | --- | --- | --- | --- | --- | --- | --- | --- | --- | --- |
| *L. santarosai* serovar Rama str. 316 - EU358063 | 0.98 | ID |  |  |  |  |  |  |  |  |  |  |
| *L. santarosai* serovar Canalzonae str. CZ188 - EU358029 | 0.97 | 0.98 | ID |  |  |  |  |  |  |  |  |  |
| *L. santarosai* serovar Guaricura str. BovG - EU357994 | 0.99 | 0.98 | 0.98 | ID |  |  |  |  |  |  |  |  |
| *L. santarosai* str. Carioca isolate M72/06-13 - KF311106 | 0.96 | 0.96 | 0.96 | 0.98 | ID |  |  |  |  |  |  |  |
| *L. santarosai* str. Bananal - KF366254 | 0.98 | 0.97 | 0.97 | 0.98 | 0.96 | ID |  |  |  |  |  |  |
| *L. santarosai* str. 2013_U280 - KP862646 | 0.96 | 0.96 | 0.96 | 0.97 | 1.00 | 0.96 | ID |  |  |  |  |  |
| *L. santarosai* str. 2013_U278 - KP862645 | 0.97 | 0.97 | 0.97 | 0.97 | 0.96 | 0.99 | 0.96 | ID |  |  |  |  |
| *L. santarosai* str. 2013_U152 - KP862632 | 0.97 | 0.97 | 0.97 | 0.97 | 0.96 | 0.99 | 0.96 | 1 | ID |  |  |  |
| Dog E – KU682052 | 0.95 | 0.94 | 0.95 | 0.96 | 0.98 | 0.95 | 0.98 | 0.96 | 0.96 | ID |  |  |
| Dog A – KX026948 | 0.95 | 0.94 | 0.95 | 0.96 | 0.98 | 0.95 | 0.98 | 0.96 | 0.96 | 1 | ID |  |
| Dog B – KX026949 | 0.95 | 0.94 | 0.95 | 0.96 | 0.98 | 0.95 | 0.98 | 0.96 | 0.96 | 1 | 1 | ID |
